# A novel quality by design approach for developing an HPLC method to analyze herbal extracts: A case study of sugar content analysis

**DOI:** 10.1371/journal.pone.0198515

**Published:** 2018-06-08

**Authors:** Jingyuan Shao, Wen Cao, Haibin Qu, Jianyang Pan, Xingchu Gong

**Affiliations:** Pharmaceutical Informatics Institute, College of Pharmaceutical Sciences, Zhejiang University, Hangzhou, China; Mizoram University, INDIA

## Abstract

The aim of this study was to present a novel analytical quality by design (AQbD) approach for developing an HPLC method to analyze herbal extracts. In this approach, critical method attributes (CMAs) and critical method parameters (CMPs) of the analytical method were determined using the same data collected from screening experiments. The HPLC-ELSD method for separation and quantification of sugars in *Codonopsis Radix* extract (CRE) samples and *Astragali Radix* extract (ARE) samples was developed as an example method with a novel AQbD approach. Potential CMAs and potential CMPs were found with Analytical Target Profile. After the screening experiments, the retention time of the D-glucose peak of CRE samples, the signal-to-noise ratio of the D-glucose peak of CRE samples, and retention time of the sucrose peak in ARE samples were considered CMAs. The initial and final composition of the mobile phase, flow rate, and column temperature were found to be CMPs using a standard partial regression coefficient method. The probability-based design space was calculated using a Monte-Carlo simulation method and verified by experiments. The optimized method was validated to be accurate and precise, and then it was applied in the analysis of CRE and ARE samples. The present AQbD approach is efficient and suitable for analysis objects with complex compositions.

## Introduction

Currently, the concept of quality by design (QbD) has been increasingly applied to the development and optimization of analytical methods, which is known as analytical quality by design (AQbD). Recently, many analytical methods were developed following an AQbD approach, such as the capillary electrophoresis method[1], Karl Fischer titration methodology[2], the supercritical fluid chromatography method[3] and so on. Compared to a traditional analytical method development approach, such as the One-Factor-At-a-time (OFAT) approach[4], the AQbD approach has several obvious advantages. Firstly, it requires less experiment time since it uses design of experiment (DOE) methods to obtain possible parameter combinations[5]. Additionally, one of the most crucial parts in the AQbD-based process is to establish an analytical design space, which is the combination of acceptable ranges of analytical parameters[6]. The obtained design space obtained can ensure the robustness of the method[7]. As defined in the ICH guideline Q8 (R2), when analytical parameters vary within the design space, the predetermined requirements for the method would still be achieved[8].

Applications of AQbD to develop an HPLC method were found in many studies[9–11]. According to published studies, the general framework of implementation of QbD for analytical method development consists of five parts. Firstly, the AQbD approach begins with the definition of the Analytical Target Profile (ATP), which mainly identifies the components to be analyzed and the required analytical technique based on the intended purpose of the method[12]. In the meantime, the critical method attributes (CMAs) are determined primarily based on prior knowledge. Many chromatographic performance criteria can be used as CMAs, such as the resolutions of critical peaks[13], the signal-to-noise ratio (SNR) of target components[14], peak symmetry[15], peak width[16], and analysis time[17]. Secondly, parameters that have a higher probability of affecting analytical results are identified through a risk assessment approach[18], DOE method[19, 20] or other methods. These parameters are known as critical method parameters (CMPs). The CMPs can be mobile phase compositions, gradient conditions, column temperature, flow rate, and so on[21, 22]. Thirdly, the quantitative relationships between CMPs and CMAs are modeled either with statistical models or with mechanism models. Fourthly, the analytical design space can be established. Fifthly, the control strategy can be set up and employed to confirm the analytical attributes of the method. Finally, method validation is necessary to demonstrate the reliability of the method.

Currently, there are not many studies on the application of the AQbD approach for developing an HPLC method for botanical extracts. A possible reason is that due to the complex composition of target compounds in herbal extracts, the gradient conditions of the HPLC method may be complicated. As a result, it is difficult to identify CMPs. On the other hand, the analytical parameters would be difficult to optimize through DOE method. Therefore, a new approach for the implementation of the QbD concept to the development of an HPLC method is still required.

In this study, a new AQbD approach of the HPLC method development for botanical extracts was represented. The content analysis of D-fructose, D-glucose, or sucrose in the *Codonopsis Radix* extracts (CREs) and the *Astragali Radix* extracts (AREs) was carried out as the example. *Codonopsis Radix* is the root of *Codonopsis pilosula*. *Astragali Radix* is the root of *Astragalus membranaceus*. Both of *Codonopsis Radix* and *Astragali Radix* are two medicinal herbs used for Shenqi Fuzheng injection, which is a botanical injection widely used as an ancillary drug for cancer clinical treatment in China[23, 24]. The CRE and the ARE were both intermediate products in the manufacture of Shenqi Fuzheng injection. The determination of sugar contents in CREs and AREs will be beneficial for quality control of Shenqi Fuzheng injection. In this study, a novel AQbD approach was recommended. Potential CMAs and potential CMPs were listed according to prior knowledge. CMAs and CMPs were determined by screening experimental data in sequence. Design space was developed after modelling with the data collected in Box-Behnken designed experiments. After the verification of design space, method validation was carried out. Finally, the newly developed method was applied to determine the sugar contents in CREs and AREs.

## Experimental

### Materials and chemicals

*Astragali Radix* collected between Jul. 2016 and Mar. 2017 and *Codonopsis Radix* collected between Sep. 2016 and Feb. 2017 were kindly provided by Limin Pharmaceutical Factory (Shaoguan, China). The batch No. of *Astragali Radix* and *Codonopsis Radix* are listed in S1 Table. No specific permissions were required for the described field studies. The locations are neither privately owned nor protected by the Chinese government. No endangered or protected species were sampled. The standard substance of D-fructose (99.5%) was purchased from Aladdin Chemistry Co., Ltd. (Shanghai, China). The standard substance of D-glucose (> 99.8%) was purchased from Sangon Biotech Co., Ltd. (Shanghai, China). The standard substance of sucrose (99%) was purchased from Sigma-Aldrich Co., Ltd. (Shanghai, China). HPLC-grade acetonitrile was obtained from Merck (Darmstadt, Germany). Triethylamine was of guaranteed reagent grade and purchased from Aladdin Chemistry Co., Ltd. (Shanghai, China). Ultrahigh-purity water was produced using a Milli-Q water purification system from Millipore (Milford, MA, USA).

### Sample preparation

Firstly, 50.0 g of *Astragali Radix* or *Codonopsis Radix* were extracted three times using a reflux extraction process with water as the extractant. Overall, 400, 300, and 300 mL of water were used for the first, second, and third extractions, respectively. The extraction time was 0.5 h for each extraction. All the obtained extracts were mixed and filtered. The AREs or CREs were then stored in a refrigerator (BL-240/241L, Shanghai Yisi Scientific Industry Co., Ltd.) before analysis. The samples of extracts were diluted with an 85% (v/v) aqueous acetonitrile. Then, the solution was centrifuged at 12,000 rpm with an Eppendorf microcentrifuge (Minispin, Eppendorf AG, Hamburg Germany) for 10 min. The supernatant was filtered through a 0.22-μm Millipore filter unit, and the filtrate was collected as a sample solution.

### HPLC analysis

All the quantitative analyses of the sugars were performed on an Agilent 1100 high-performance liquid chromatography system (Agilent Technologies, Palo Alto, CA, USA). The analytes were detected by an Alltech 2000ES ELSD. The separations were carried out on a Waters XBridge Amide column (4.6×250 mm, 5 μm, Waters, Milford, MA, USA). The samples and standards were separated with linear gradient elution. The mobile phase was composed of solvent A (an appropriate amount of triethylamine in water) and solvent B (an appropriate amount of triethylamine in acetonitrile). In addition, there was a column wash of 60% B in mobile phase for 10 min after each run and column equilibration with initial mobile phase composition for 10 min. A mixed standard stock solution containing D-fructose, D-glucose and sucrose was prepared. Standard solutions with other concentrations were prepared by diluting the stock solution with 85% (v/v) aqueous acetonitrile. All standards were filtered through 0.22-μm Millipore membranes before analysis. The injection volume of samples or standards was 5 μL. The ELSD impactor was set to OFF mode. The gain value was fixed at 1 during all the experiments. Calibration curves were established, and quantitative analyses of samples were based on the calibration plots of the logarithm of peak areas versus the logarithm of concentrations for each sugar.

### Experimental design

Sugar analysis can be performed using an isocratic elution system of acetonitrile-water[25, 26]. However, a linear gradient elution system of acetonitrile-water may lead to better resolutions with a shorter analysis time[27]. Therefore, a linear gradient was adopted in this work.

Some separation and detection factors were investigated, including initial solvent B content in mobile phase (X_1_), final solvent B content in mobile phase (X_2_), the flow rate of the mobile phase (X_3_), column temperature (X_4_), gradient run time (X_5_), the proportion of triethylamine in the mobile phase (X_6_), ELSD drift tube temperature (X_7_), and flow rate of nitrogen gas (X_8_). The linear gradient elution was conducted as follows: t: 0-X_5_ (min), B%: X_1_-X_2_ (%). The coded and uncoded values of each parameter are shown in Table 1. A two-level fractional designed experiment with three center points was utilized to analyze the effects of these eight parameters on the analytical results, as shown in Table 2.

**Table 1:** Parameters and their levels for two-level fractional design.

Analytical parameter	Symbol	Unit	Coded variable		
			-1	0	1
Initial solvent B content in the mobile phase	X_1_	%	83	85	87
Final solvent B content in the mobile phase	X_2_	%	76	78	80
Flow rate	X_3_	mL/min	0.8	0.9	1.0
Column temperature	X_4_	°C	30	35	40
Gradient run time	X_5_	min	34	37	40
The proportion of triethylamine in the mobile phase	X_6_	% (v,v)	0.2	0.3	0.4
Drift tube temperature	X_7_	°C	95	100	105
Flow rate of nitrogen gas	X_8_	L/min	1.6	1.8	2.0

**Table 2:** The conditions and results of two-level fractional designed experiments.

Run	Analytical parameters								Potential CMAs					
	X_1_ (%)	X_2_ (%)	X_3_ (mL/min)	X_4_ (°C)	X_5_ (min)	X_6_ (%)	X_7_ (°C)	X_8_ (L/min)	Y_1_ (min)	Y_2_	Y_3_ (min)	Y_4_ (min)	Y_5_	Y_6_
1	83	76	0.8	30	34	0.2	95	1.6	16.00	77.18	24.68	1.68	131.2	2.31
2	87	76	0.8	30	34	0.4	105	2.0	20.56	72.32	30.87	1.45	129.3	3.32
3	83	80	0.8	40	34	0.4	95	2.0	15.45	46.95	25.86	1.43	216.2	2.29
4	87	80	0.8	40	34	0.2	105	1.6	20.01	59.51	33.30	1.43	131.6	2.55
5	83	76	0.8	40	40	0.4	105	1.6	14.91	45.30	23.55	1.25	205.8	2.40
6	87	76	0.8	40	40	0.2	95	2.0	19.26	52.14	30.43	1.54	137.0	2.46
7	83	80	0.8	30	40	0.2	105	2.0	16.87	60.66	28.60	1.84	73.98	3.00
8	87	80	0.8	30	40	0.4	95	1.6	22.42	61.93	37.60	1.77	145.3	2.89
9	83	76	1.0	40	34	0.2	105	2.0	11.79	30.03	18.77	1.18	259.6	2.21
10	87	76	1.0	40	34	0.4	95	1.6	15.39	37.23	24.56	1.59	210.4	2.08
11	83	80	1.0	30	34	0.4	105	1.6	13.48	34.42	22.76	1.65	173.8	2.75
12	87	80	1.0	30	34	0.2	95	2.0	39.59	55.32	30.31	1.58	111.9	2.68
13	83	76	1.0	30	40	0.4	95	2.0	13.26	28.39	21.30	1.83	126.7	2.47
14	87	76	1.0	30	40	0.2	105	1.6	17.35	52.65	27.95	1.51	204.3	2.90
15	83	80	1.0	40	40	0.2	95	1.6	12.29	26.51	21.17	1.30	232.7	2.10
16	87	80	1.0	40	40	0.4	105	2.0	16.57	38.48	29.13	1.82	210.0	2.44
17	85	78	0.9	35	37	0.3	100	1.8	16.36	53.53	27.07	1.34	205.1	2.65
18	85	78	0.9	35	37	0.3	100	1.8	16.37	55.98	27.04	1.43	206.9	2.69
19	85	78	0.9	35	37	0.3	100	1.8	16.35	56.06	27.10	1.34	188.5	2.73

After preliminary experiments, some separation and detection parameters were fixed as follows: gradient run time of 37 min, the proportion of triethylamine in the mobile phase of 0.3%, drift tube temperature of 100 °C, and flow rate of nitrogen gas of 1.8 L/min. A Box-Behnken design with five center points was then used to evaluate the quantitative relationships between the CMPs and the CMAs, as shown in Table 3.

**Table 3:** The conditions and results of the Box-Behnken designed experiments.

Run	CMPs				CMAs		
	X_1_ (%)	X_2_ (%)	X_3_ (mL/min)	X_4_ (°C)	Y_1_ (min)	Y_2_	Y_3_ (min)
1	83	76	0.9	35	13.91	44.16	21.71
2	87	76	0.9	35	17.97	53.00	28.15
3	83	80	0.9	35	14.45	50.54	24.46
4	87	80	0.9	35	18.93	71.15	31.39
5	85	78	0.8	30	18.91	77.60	29.87
6	85	78	1.0	30	15.44	55.39	25.67
7	85	78	0.8	40	17.20	62.01	27.91
8	85	78	1.0	40	13.92	50.04	22.85
9	83	78	0.9	30	14.86	58.91	24.13
10	87	78	0.9	30	19.38	64.54	31.05
11	83	78	0.9	40	13.47	30.08	21.79
12	87	78	0.9	40	17.67	60.76	29.18
13	85	76	0.8	35	17.64	68.05	27.45
14	85	80	0.8	35	18.52	70.99	31.02
15	85	76	1.0	35	14.14	45.49	22.39
16	85	80	1.0	35	14.98	47.98	26.09
17	83	78	0.8	35	15.90	53.78	25.89
18	87	78	0.8	35	20.14	50.40	32.23
19	83	78	1.0	35	12.44	39.86	19.75
20	87	78	1.0	35	16.89	52.90	27.78
21	85	76	0.9	30	16.64	67.42	26.40
22	85	80	0.9	30	17.46	62.03	29.32
23	85	76	0.9	40	15.17	53.00	24.13
24	85	80	0.9	40	15.71	52.81	27.34
25	85	78	0.9	35	16.22	57.21	26.86
26	85	78	0.9	35	16.09	52.71	26.37
27	85	78	0.9	35	16.09	57.83	25.85
28	85	78	0.9	35	16.10	58.39	26.57
29	85	78	0.9	35	16.11	46.03	26.27

### Data processing

To estimate which parameters were significant for responses, the standard partial regression coefficient method was used to analyze the results of two-level fractional design and select CMPs[28, 29]. Firstly, the response values were standardized according to Eq (1).

$${Y_{i}}^{'}=\frac{Y_{i}-\overset{-}{Y}}{{SD}_{i}}\;(i=1,\;2,\;3)$$ (1)

where Yi', Y_i_ and $\bar{\text{Y}}$ represent the standardized value, the measured value, and the average value of each response, respectively; SD_i_ is the standard deviation of each response; and number i (i = 1, 2, 3) represents retention time of the D-glucose peak in CRE samples, SNR value of the D-glucose peak in CRE samples, and retention time of the sucrose peak in ARE samples, respectively. Multiple linear regression analysis was then used to calculate standard partial regression coefficients according to Eq (2).

$$Y_{i}^{\prime }=a_{0,i}+\sum_{j=1}^{8}a_{j,i}X_{j}\;\left(\text{j}=1,\;2,\ldots ,\;8\right)$$ (2)

where a_0,i_ is a constant; X_j_ represents potential CMPs; and a_j,i_ is the standard partial regression coefficient. The absolute values of each a_j,i_ were weighted and summed up to evaluate the total influences of each parameter X_j_ on all the responses, as seen in Eq (3).

$$A_{j}=\sum_{i=1}^{3}w_{i}\left|a_{j,i}\right|$$ (3)

where A_j_ is named as importance factor. Parameters with higher A_j_ values were expected to have greater influences on responses. In this study, each response was considered equally important, which means that the w_i_ values of each coefficient were 1/3, respectively.

By applying multivariate regression analysis, quadratic models were built to obtain the quantitative models between CMPs and CMAs according to Eq (4).

$$Y=b_{0}+\sum_{i=1}^{4}b_{i}X_{i}+\sum_{i=1}^{4}b_{ii}X_{i}^{2}+\sum_{i=1}^{3}\sum_{j=i+1}^{4}b_{ij}X_{i}X_{j}$$ (4)

where b_0_ is a constant; and b_i_, b_ii_ and b_ij_ represent the regression coefficients for linear, quadratic, and interaction terms, respectively. The analysis of the results was performed using Design Expert (version 8.0.6, Stat-Ease Inc., USA).

Based on the specific goals of CMAs, a Monte-Carlo method was performed using an in-house MATLAB program (R2016a, Version 9.0, The MathWorks Inc., USA) to calculate the design space[30]. The detailed calculation processes were described in previous work[31]. A brief description is given as follows. It is assumed that the experimental results were subject to a normal distribution. The mean value of the normal distribution was assumed to be the measured response value. The relative standard deviation (RSD) of the normal distribution was assumed to be the same as that of the center points in the Box-Behnken designed experiments. Random response values were then obtained and modeled by Eq (4) through every simulation. The prediction values of CMAs were obtained using models built with random response values. The probability to meet all the analytical goals was then calculated based on the model prediction results. The design space was defined with the probability higher than 0.90. The simulation was used 10,000 times to obtain the probability-based design space. In the Monte-Carlo simulation, coded values of variables were used.

### Method validation

After the design space was obtained, an operating point with a high probability to attain CMA goals was chosen for the optimized analytical method. Then, method validation experiments were carried out for ARE and CRE samples respectively, including tests of linearity and sensitivity, analytical precision, stability and accuracy. The limit of detection (LOD) and the limit of quantification (LOQ) were determined by SNR values at 3:1 and 10:1, respectively. The same sample solution was injected for six times continuously to evaluate injection precision. Six sample solutions were prepared in parallel and tested during a single day for intra-day precision. Inter-day precision was evaluated by analyzing replicate samples for three consecutive days, respectively. The stability of a sample solution was evaluated at regular intervals for 24 h, including measurements at 0, 2, 4, 6, 8, 10, 12, 24 h. To evaluate the method accuracy, recovery experiments were performed in triplicate at concentration levels of 50, 100, and 150 percent of the sample solution. Certain amounts of sugars were added to the sample solution and then analyzed. The recovery was calculated using the ratio of the measured contents of each sugar to added contents. All those results were evaluated by RSD values of the peak areas or the contents of corresponding components.

## Results and discussion

### The novel AQbD approach for herbal extracts

Differed from the conventional AQbD approach described in the introduction section, CMAs and CMPs were determined in sequence with a same data set in the novel AQbD approach. Therefore, the present approach can be more suitable and efficient when dealing with complex systems. Botanical extracts are usually mixtures with many unknown ingredients. When the analytical parameters change, peak separation may be dramatically affected. Therefore, it is difficult to identify CMAs in the initial stage of HPLC method development. At most occasions, potential CMAs could be determined based on prior knowledge. CMAs could be further determined based on some experimental results. If these experiments were also used for CMP selection, CMAs and CMPs can be determined using a same set of experimental data, which will be very efficient.

### CMA and CMP identification

**Fig 1:**
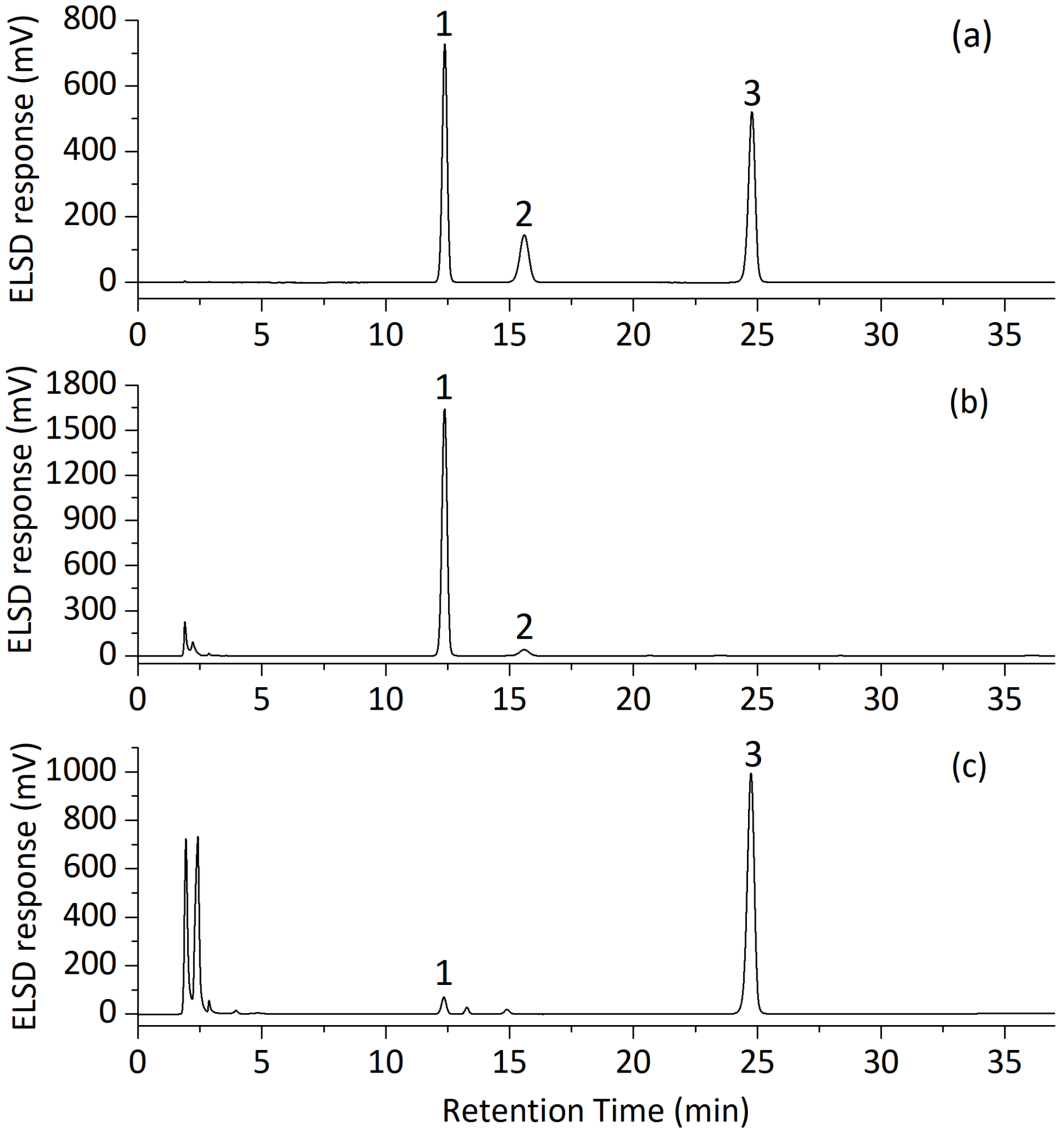
Typical HPLC-ELSD chromatograms for standards and sample solutions. (a) Standard solution; (b) CRE samples; (c) ARE samples. Peaks 1, 2, 3 represented D-fructose, D-glucose and sucrose, respectively.

Typical chromatograms of standards and samples are shown in Fig 1. CRE samples were found to contain two sugar peaks of D-fructose and D-glucose in this study. Xu et al.[32] reported that ARE samples mainly contained two sugars, including D-fructose and sucrose, according to results of thin-layer chromatography, HPLC and GC-MS analysis. In this study, a similar conclusion could be made.

Some chromatographic performance criteria must be considered, including the resolution between adjacent peaks, the SNR of the target components, analysis time, and so on. The resolution between adjacent peaks is the measurement of the HPLC separation performance. The resolution value above 1.5 generally indicates that good separation occurs between adjacent peaks. The SNR of a chromatographic peak is an important system suitability parameter that can be an accurate reflection of the sensitivity performance of the detector. Peak symmetry and peak width are used to describe the peak shape that have a certain relationship with the accuracy of the quantitative results. In addition, the analysis time is commonly used as a CMA in the method development process because a shorter run time is usually favored. The retention time of the last peak is used to represent the method run time.

In this study, some of these criteria were studied as potential CMAs, including the retention time of the D-glucose peak in CRE samples (Y_1_), the SNR value of the D-glucose peak in CRE samples (Y_2_), the retention time of the sucrose peak in ARE samples (Y_3_), the width of D-glucose peak in CRE samples (Y_4_), the SNR value of D-fructose in ARE samples (Y_5_), and the resolution between the D-fructose peak and its subsequent peak in ARE samples (Y_6_). The index values of potential CMAs were collected in the two-level fractional designed experiments, as shown in Table 2.

In Table 2, some of the criteria met the analytical requirements for all the experiments, which indicates that those criteria were not CMAs. For example, the resolution values between the D-fructose peak and its subsequent peak in ARE samples were all higher than 1.50, which means that satisfactory separation was achieved. The SNR value of D-fructose in ARE samples was higher than 70, whereas the SNR value of D-glucose in the *Codonopsis Radix* sample varied significantly according to analytical conditions, from 26.51 to 77.18. Since the peak area of D-glucose in the *Codonopsis Radix* sample was much smaller compared to other components, the SNR value of that peak was taken as a CMA. In addition, the retention time of the D-glucose peak in the CRE samples and the retention time of the sucrose peak in the ARE samples were used as CMAs to reduce the analysis time.

Because potential CMAs included resolution, signal-to-noise ratio, peak width, and retention time, a total of 8 potential CMPs covering chromatographic operation and detection were selected based on prior knowledge in this work. The initial and final solvent B content in the mobile phase, the proportion of triethylamine in the mobile phase, gradient run time, column temperature, and flow rate were considered to be potential CMPs for better separation of the target sugars. The ELSD parameters, including drift tube temperature and flow rate of nitrogen gas were considered as potential CMPs for better detectability.

The results of multiple linear regression analysis and the normalized regression coefficients of each response are shown in Table 4. Importance factor values are also listed in Table 4. The analysis parameters with the A_j_ value ranked in the top four of all the eight potential CMPs were selected as CMPs. It was concluded that initial solvent B content in the mobile phase (X_1_), final solvent B content in the mobile phase (X_2_), flow rate of the mobile phase (X_3_), and column temperature (X_4_) were CMPs.

**Table 4:** Results of the multiple linear regression analysis.

Term	Y_1_	Y_2_	Y_3_	A_j_ value
X_1_	0.61	0.36	0.81	0.59
X_2_	0.30	-0.051	0.37	0.24
X_3_	-0.062	-0.78	-0.55	0.46
X_4_	-0.36	-0.48	-0.24	0.36
X_5_	-0.21	-0.21	0.12	0.18
X_6_	-0.23	-0.22	5.688×10^−4^	0.15
X_7_	-0.24	0.035	-0.014	0.096
X_8_	0.23	-0.047	-4.139×10^−3^	0.092
R^2^	0.6761	0.8903	0.9850	—

### Effects of CMPs on CMAs

The results of the Box-Behnken experiments are shown in Table 3. The regression coefficients, P values, and R^2^ values of regression models between CMPs and CMAs are listed in Table 5. For each CMA, R^2^ value that was more than 0.80 indicates that most variations of experimental data can be explained. According to the P values, the linear effects of initial solvent B content in the mobile phase, the flow rate, and column temperature were significant for all three CMAs. Final solvent B content in the mobile phase significantly affected two CMAs of the retention time of D-glucose peak in CRE samples and the retention time of sucrose peak in ARE samples. The interaction term between the initial and final solvent B content in the mobile phase was significant for the retention time of D-glucose peak in CRE samples.

**Table 5:** ANOVA results for multiple regression models.

Term	Y_1_		Y_2_		Y_3_	
	Coefficient	P value	Coefficient	P value	Coefficient	P value
Constant	16.12		54.43		26.38	
X_1_	2.16	< 0.0001**	6.29	0.0038**	3.50	< 0.0001**
X_2_	0.38	< 0.0001**	2.03	0.2818	1.62	< 0.0001**
X_3_	-1.71	< 0.0001**	-7.60	0.0009**	-2.49	< 0.0001**
X_4_	-0.80	< 0.0001**	-6.43	0.0032**	-1.10	< 0.0001**
X_1_X_2_	0.11	0.0059**	2.94	0.3652	0.12	0.4459
X_1_X_3_	0.050	0.1466	4.11	0.2122	0.42	0.0154*
X_1_X_4_	-0.081	0.0265*	6.26	0.0661	0.12	0.4552
X_2_X_3_	-0.011	0.7428	-0.12	0.9712	0.034	0.8273
X_2_X_4_	-0.072	0.0465*	1.30	0.6850	0.072	0.6463
X_3_X_4_	0.047	0.1767	2.56	0.4289	-0.21	0.1860
X_1_^2^	0.12	0.0004**	-4.41	0.0955	-0.084	0.4981
X_2_^2^	0.056	0.0461*	2.66	0.2999	0.21	0.1056
X_3_^2^	0.13	0.0002**	1.14	0.6507	0.091	0.4622
X_4_^2^	0.096	0.0023**	3.65	0.1610	0.18	0.1516
R^2^	0.9994		0.8079		0.9952	
P value	< 0.0001		0.0056		< 0.0001	

* p<0.05

** p<0.01

The contour plots were obtained to analyze the effects of CMPs on CMAs, as shown in S1–S3 Figs. It was inferred from S1 Fig that a lower initial and final solvent B content in mobile phase would result in a shorter retention time of D-glucose peak in CRE samples. Increasing water content of mobile phase would reduce the retention time of the sugar peak. The retention time of D-glucose peak would also be decreased by increasing the flow rate and column temperature. The same conclusion could be drawn for the retention time of the sucrose peak in ARE samples according to S3 Fig. However, a faster flow rate and a higher column temperature would result in a lower SNR value of the D-glucose peak in CRE samples, as shown in S2 Fig.

### Design space development and operating point selection

**Fig 2:**
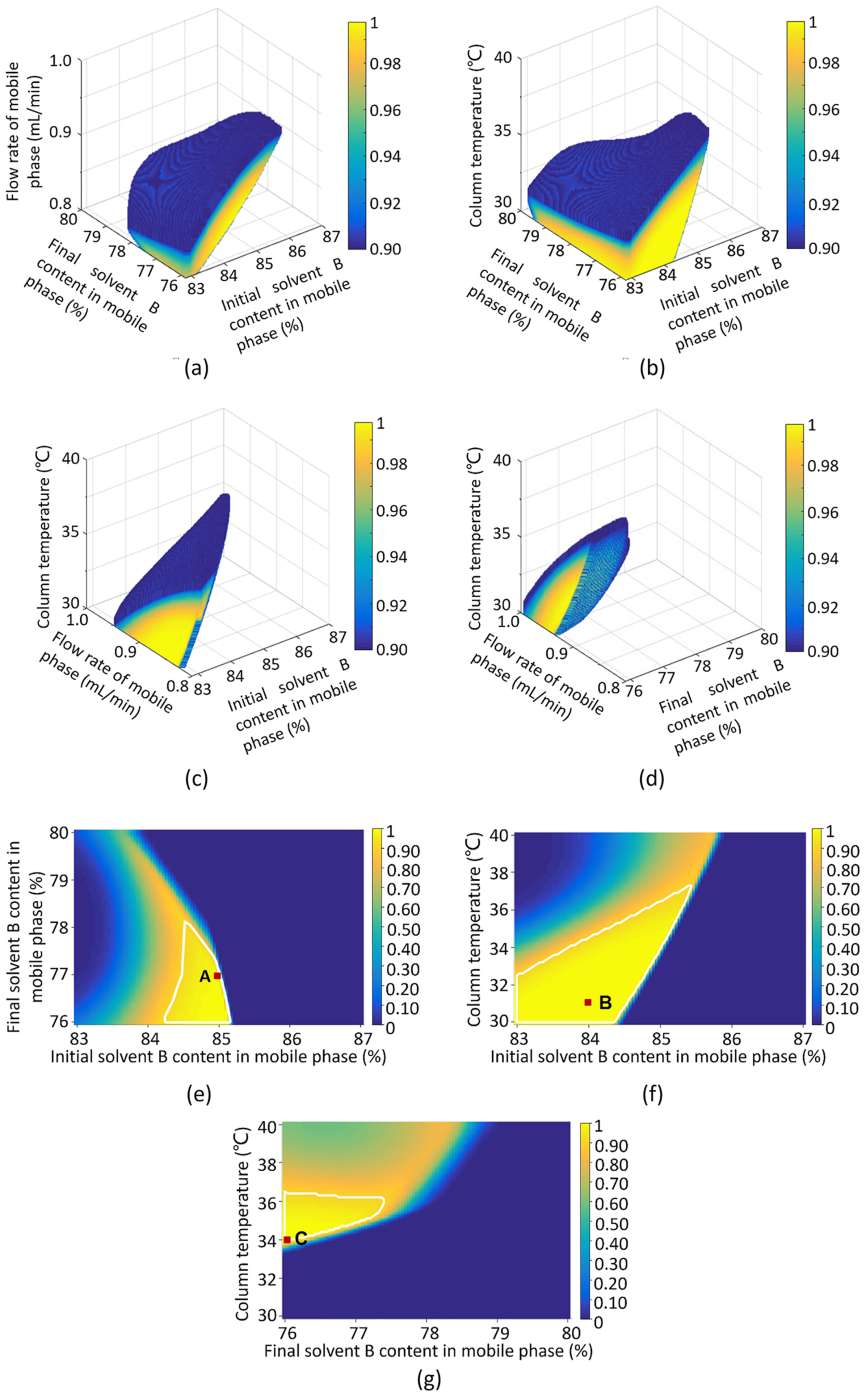
Analytical design space and verification points. (a) Column temperature = 35 °C; (b) Flow rate of mobile phase = 0.9 mL/min; (c) Final solvent B content in the mobile phase = 76%; (d) Initial solvent B content in the mobile phase = 85%; (e) Flow rate of mobile phase = 0.9 mL/min; column temperature = 35 °C; (f) Final solvent B content in the mobile phase = 76%; flow rate of mobile phase = 0.9 mL/min; (g) Initial solvent B content in the mobile phase = 85%; flow rate of mobile phase = 0.9 mL/min. Color bar refers to the probability to attain CMA goals; and the probability of areas surrounded by white borders to attain CMA goals was higher than 0.90.

To obtain optimum chromatographic results, the design spaces were calculated based on the specific goals of each CMA. Since shorter analysis time was required, the upper limits of the retention time of D-glucose peak and the retention time of the sucrose peak were set at 16 and 26 min, respectively. Meanwhile, a higher SNR value for a chromatographic peak was an important reflection of the analytical sensitivity. Therefore, the lower limit of the SNR value of the D-glucose peak in CRE samples was set at 50. Then, the Monte-Carlo method was performed to calculate the design spaces with a probability above 0.90 to attain CMA limits. The design spaces obtained were irregular regions, as shown in Fig 2(a)–2(d).

For the accuracy of chromatographic conditions, three combinations of appropriate analysis conditions with high probabilities to attain CMA goals were selected and applied to the method verification experiments. The combined values of each CMP are presented in Table 6, and the other fixed HPLC parameters were the same as the Box-Behnken designed experiments. These three methods were named A, B, and C, respectively. The verification points in the design spaces are shown in Fig 2(e)–2(g). Verification results are listed in Table 6. The experimental values of the retention time of the D-glucose peak in CRE samples and the retention time of the sucrose peak in ARE samples were roughly consistent with the predicted values. Remarkable differences between the predicted values and the experimental values for the SNR values of D-glucose peak in CRE samples were observed. This result likely occurred due to the lower R^2^ value of the model used for prediction.

**Table 6:** Verification conditions and results.

Verification condition	A	B	C
The probability to attain CMA goals	0.97	1	0.99
Initial solvent B content in mobile phase (%)	85	84	85
Final solvent B content in mobile phase (%)	77	76	76
Flow rate (mL/min)	0.9	0.9	0.9
Column temperature (°C)	35	31	34
Experimental value of Y_1_	16.40	15.59	16.37
Predicted value of Y_1_	15.94	15.41	15.94
Experimental value of Y_2_	75.26	69.47	75.67
Predicted value of Y_2_	57.94	61.09	59.23
Experimental value of Y_3_	26.46	24.74	26.08
Predicted value of Y_3_	25.74	24.06	25.15

### HPLC-ELSD method verification

The analytical conditions of method C were finally used to determine the sugar concentrations and method validation experiments were carried out. The conditions were shown as follows. Initial solvent B content in the mobile phase was 85%. Final solvent B content in the mobile phase was 76%. Flow rate of mobile phase was 0.9 mL/min. Column temperature was 34 °C. Gradient run time was 37 min. The proportion of triethylamine in the mobile phase was 0.3%. Drift tube temperature was 100 °C. Flow rate of nitrogen gas was 1.8 L/min.

The linearity of the method was confirmed by establishing calibration curves for each sugar. The calibration curve equations and their corresponding determination coefficients, analytical ranges, the limits of detection and the limits of quantification are listed in Table 7. The correlation coefficient values R >0.9990 indicate a high-level of linearity for each sugar. The results of analytical precision, stability and accuracy of this method are summarized in Table 8. All the RSD values were less than 5%, which indicates that the precision of the method could meet the requirement of analysis, and the sample solutions were stable within 24 h. The average recovery of sugars at three concentration levels ranged from 95.4% to 105.4%, whereas the RSD value ranged from 1.69% to 3.65%, which indicated the method had good accuracy.

**Table 7:** Linearity and analytical range.

Component	Calibration curve equation	R	Analytical range (mg/mL)	LOD (mg/mL)	LOQ (mg/mL)
D-Fructose	y = 1.4866x + 6.7358	0.9990	0.3551–2.5688	0.0217	0.0639
D-Glucose	y = 1.6239x + 6.7301	0.9997	0.1162–0.8408	0.0349	0.0697
Sucrose	y = 1.4680x + 6.7636	0.9996	0.3512–2.5408	0.0197	0.0393

**Table 8:** Results of analytical precision, sample stability and accuracy experiments.

Component	Injection precision	Intra-day precision	Inter-day precision	Sample stability	Recovery	
	RSD (%)	RSD (%)	RSD (%)	RSD (%)	Average value (%)	RSD (%)
D-Fructose in CRE samples	3.82	1.59	2.51	4.45	102.4	3.65
D-Glucose in CRE samples	3.73	2.86	0.16	4.92	105.4	2.65
D-Fructose in ARE samples	3.22	0.58	3.48	3.62	96.7	3.11
Sucrose in ARE samples	2.20	0.81	3.79	2.29	95.4	1.69

### Application of the method

Fifteen batches of *Codonopsis Radix* and fifteen batches of *Astragali Radix* were extracted with water as the extractant. The developed HPLC-ELSD method was applied to determine the sugar concentrations in aqueous extract solutions of *Codonopsis Radix* and *Astragali Radix*. The results are listed in S1 Table. The D-fructose concentrations and D-glucose concentrations of CRE samples were 1.502–2.189 mg/mL and 0.123–0.432 mg/mL, respectively. The D-fructose concentration and sucrose concentration of ARE samples were 0.041–0.179 mg/mL and 0.812–2.475 mg/mL, respectively.

## Conclusion

The present paper describes a novel AQbD approach to develop robust analytical methods. In this approach, data collected from screening experiments were used to determine CMAs and CMPs in sequence. The development of the HPLC-ELSD method for the quantification of the sugar concentrations in extract solutions of *Codonopsis Radix* and *Astragali Radix* was used as a sample. Potential CMAs and potential CMPs were obtained after analytical target profiling. A two-level fractional designed experiment was employed to select the CMPs and CMAs. Three CMAs of retention time of the D-glucose peak of CRE samples, the SNR of D-glucose peak of CRE samples, and the retention time of the sucrose peak in ARE samples were determined. Four CMPs of initial and final solvent B content in the mobile phase, flow rate, and column temperature were also found with a standard partial regression coefficient method. After the Box-Behnken experiments, the quantitative models between CMPs and CMAs were successfully constructed. The design space was then calculated using a Monte-Carlo simulation method. The design space was also verified. A set of analytical conditions with a high probability to attain CMA goals was recommended, including initial solvent B content in the mobile phase of 85%, final solvent B content in the mobile phase of 76%, flow rate of mobile phase of 0.9 mL/min, and column temperature of 34°C. The developed method was validated successfully and applied to simultaneously determine the contents of D-fructose, D-glucose and sucrose in CRE and ARE samples.

## Supporting information

S1 Table Sugar concentrations in different extract solutions. (PDF)

S1 Fig Contour plot of the retention time of glucose peak in *Codonopsis Radix* extract samples. (a) Flow rate of mobile phase = 0.9 mL/min; column temperature = 35°C; (b) Final solvent B content in the mobile phase = 78%; column temperature = 35°C; (c) Final solvent B content in the mobile phase = 78%; flow rate of mobile phase = 0.9 mL/min; (d) Initial solvent B content in the mobile phase = 85%; column temperature = 35°C; (e) Initial solvent B content in the mobile phase = 85%; flow rate of mobile phase = 0.9 mL/min; (f) Initial solvent B content in the mobile phase = 85%; final solvent B content in the mobile phase = 78%.
(PDF)

S2 Fig Contour plot of the SNR value of glucose peak in Codonopsis Radix extract samples. (a) Flow rate of mobile phase = 0.9 mL/min; column temperature = 35°C; (b) Final solvent B content in the mobile phase = 78%; column temperature = 35°C; (c) Final solvent B content in the mobile phase = 78%; flow rate of mobile phase = 0.9 mL/min; (d) Initial solvent B content in the mobile phase = 85%; column temperature = 35°C; (e) Initial solvent B content in the mobile phase = 85%; flow rate of mobile phase = 0.9 mL/min; (f) Initial solvent B content in the mobile phase = 85%; final solvent B content in the mobile phase = 78%.
(PDF)

S3 Fig Contour plot of the retention time of sucrose peak in *Astragali Radix* extract samples. (a) Flow rate of mobile phase = 0.9 mL/min; column temperature = 35°C; (b) Final solvent B content in the mobile phase = 78%; column temperature = 35°C; (c) Final solvent B content in the mobile phase = 78%; flow rate of mobile phase = 0.9 mL/min; (d) Initial solvent B content in the mobile phase = 85%; column temperature = 35°C; (e) Initial solvent B content in the mobile phase = 85%; flow rate of mobile phase = 0.9 mL/min; (f) Initial solvent B content in the mobile phase = 85%; final solvent B content in the mobile phase = 78%.
(PDF)
