# Supplementary material for: A novel quality by design approach for developing an HPLC method to analyze herbal extracts: A case study of sugar content analysis
Source: PLoS One. 2018 Jun 8;13(6):e0198515. doi: 10.1371/journal.pone.0198515 (PMC5993237; doi:10.1371/journal.pone.0198515)
Supplement: S1 Table — (PDF) [file pone.0198515.s001.pdf]

S1 Table. Sugar concentrations in different extract solutions.

Aqueous extract solutions	Batch No.	Sugar concentration (mg/mL)		
		D-Fructose	D-Glucose	Sucrose
CRE sample 1	160914	2.189	0.432	—
CRE sample 2	170206	1.698	0.303	—
CRE sample 3	160912	1.979	0.324	—
CRE sample 4	160910	2.024	0.316	—
CRE sample 5	170103	1.751	0.355	—
CRE sample 6	170205	1.660	0.278	—
CRE sample 7	170101	1.769	0.375	—
CRE sample 8	160913	2.033	0.311	—
CRE sample 9	170104	1.502	0.248	—
CRE sample 10	170203	2.107	0.296	—
CRE sample 11	170102	1.560	0.123	—
CRE sample 12	160911	2.054	0.307	—
CRE sample 13	170207	1.884	0.290	—
CRE sample 14	170105	1.962	0.362	—
CRE sample 15	170204	1.804	0.255	—
ARE sample 1	Unknown	0.060	—	2.475
ARE sample 2	Unknown	0.054	—	1.842
ARE sample 3	Unknown	0.050	—	2.179
ARE sample 4	Unknown	0.045	—	2.157
ARE sample 5	Unknown	0.179	—	0.812
ARE sample 6	170301	0.058	—	2.460
ARE sample 7	170302	0.055	—	2.242
ARE sample 8	Unknown	0.074	—	1.753
ARE sample 9	Unknown	0.044	—	2.055
ARE sample 10	161221	0.123	—	1.245
ARE sample 11	Unknown	0.055	—	2.308
ARE sample 12	Unknown	0.041	—	1.769
ARE sample 13	160715	0.066	—	1.984
ARE sample 14	161225	0.054	—	2.209
ARE sample 15	Unknown	0.042	—	2.389
