# Supplementary material for: A novel quality by design approach for developing an HPLC method to analyze herbal extracts: A case study of sugar content analysis
Source: PLoS One. 2018 Jun 8;13(6):e0198515. doi: 10.1371/journal.pone.0198515 (PMC5993237; doi:10.1371/journal.pone.0198515)
Supplement: S3 Fig — (a) Flow rate of mobile phase = 0.9 mL/min; column temperature = 35°C; (b) Final solvent B content in the mobile phase = 78%; column temperature = 35°C; (c) Final solvent B content in the mobile phase = 78%; flow rate of mobile phase = 0.9 mL/min; (d) Initial solvent B content in the mobile phase = 85%; column temperature = 35°C; (e) Initial solvent B content in the mobile phase = 85%; flow rate of mobile phase = 0.9 mL/min; (f) Initial solvent B content in the mobile phase = 85%; final solvent B content in the mobile phase = 78%. (PDF) [file pone.0198515.s004.pdf]

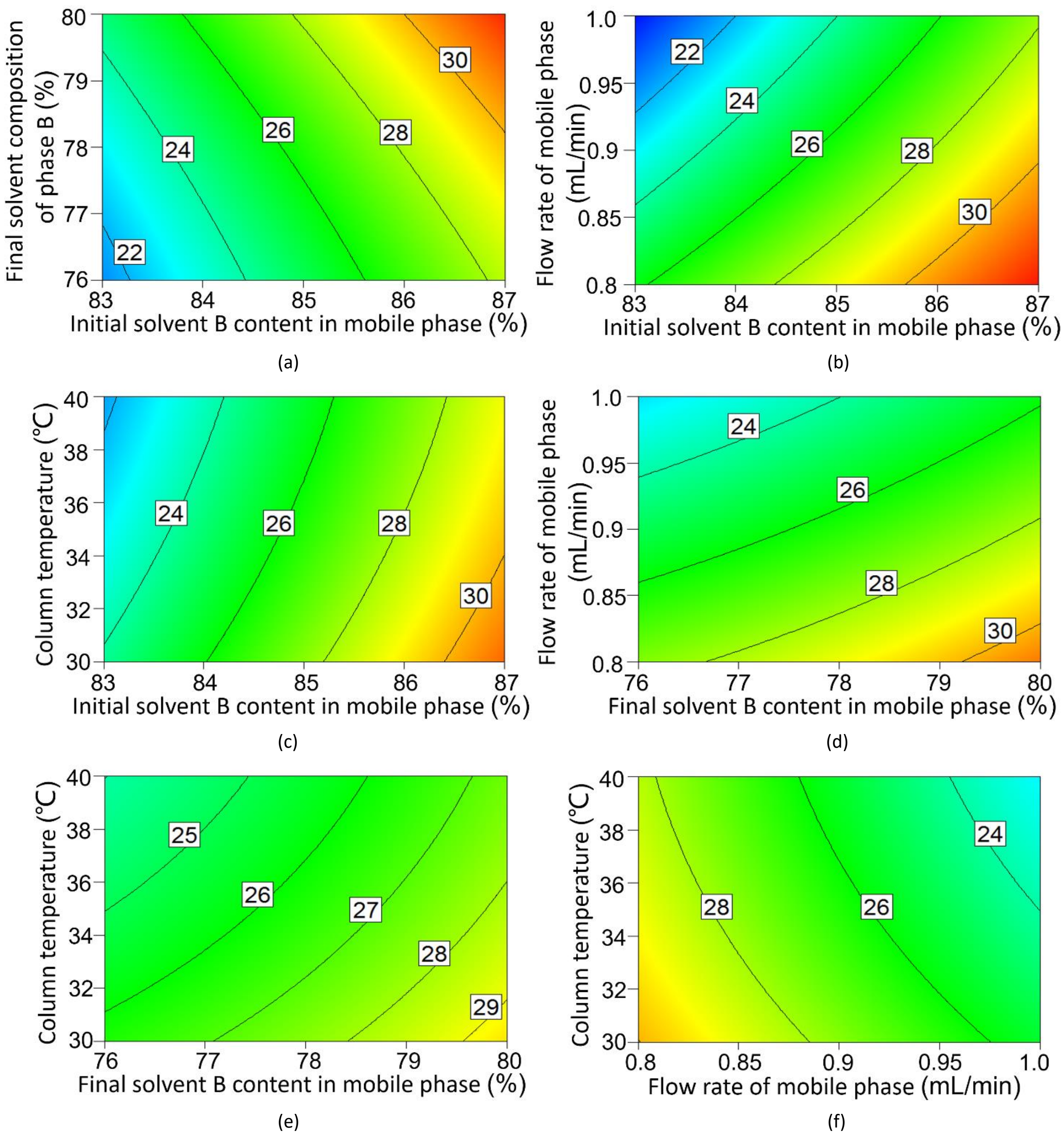

S3 Fig. Contour plot of the retention time of sucrose peak in *Astragali Radix* extract samples. (a) Flow rate of mobile phase = 0.9 mL/min; column temperature = 35℃; (b) Final solvent B content in the mobile phase = 78%; column temperature = 35℃; (c) Final solvent B content in the mobile phase = 78%; flow rate of mobile phase = 0.9 mL/min; (d) Initial solvent B content in the mobile phase = 85%; column temperature = 35℃; (e) Initial solvent B content in the mobile phase = 85%; flow rate of mobile phase = 0.9 mL/min; (f) Initial solvent B content in the mobile phase = 85%; final solvent B content in the mobile phase = 78%.
